# Effect of Steam Pressure Toasting Duration on Snowbird Faba Bean Seeds and the Impact on the Intestinal and Metabolic Characteristics in Dairy Cows

**DOI:** 10.3390/ani14030483

**Published:** 2024-02-01

**Authors:** María E. Rodríguez Espinosa, Víctor H. Guevara-Oquendo, Jiangfeng He, Weixiang Zhang, Peiqiang Yu

**Affiliations:** 1 Department of Animal and Poultry Science, College of Agriculture and Bioresources, University of Saskatchewan, Saskatoon, SK S7N 5A8, Canada; 2 Inner Mongolia Academy of Agriculture and Husbandry Science, Hohhot 010031, China; 3 College of Animal Science and Technology, Henan University of Animal Husbandry and Economy, Zhengzhou 450046, China

**Keywords:** dairy cows, faba bean, thermal processing, intestinal digestibility, protein supply, steam pressure toasting

## Abstract

**Simple Summary:**

The response of feedstuffs to thermal processing depends on the type of feed and the thermal processing methods being applied. Steam pressure toasting (SPT) has been used recently to modify the nutrient degradability and enhance the nutritional quality of pulses. This study aimed to determine the effects of SPT duration in faba bean seeds (FBS) on the intestinal and metabolic characteristics of dairy cows. Our results show that SPT increased the nutrient values of FBS in terms of total metabolizable protein (MP), truly absorbed nutrient supply (DVE), and feed milk value (FMV).

**Abstract:**

The response of feedstuffs to thermal processing depends on the type of feed and the thermal processing methods being applied. Steam pressure toasting (SPT) has been used to modify the nutrient degradability and enhance the nutritional quality of pulses, including faba bean seeds (FBS). Strategic feeding approaches are essential for balancing diets and maintaining adequate nutrition, especially in high-performing ruminants. This research aimed to determine the effects of SPT duration in FBS on the intestinal and metabolic characteristics of dairy cows. Faba Bean seeds (three harvesting years) were processed at 121 °C for 0, 30, 60, 90, and 120 min. Rumen degradation and intestinal digestion were determined using the in situ and modified in vitro three-step techniques. The true protein supplied to the small intestine was also determined using the NRC and DVE systems. Our results showed a reduced total digested DM (TDDM) with longer SPT duration (quadratic, *p* = 0.02). The intestinally digested crude protein (IADP) increased from 62 to 220 g/kg DM with 0 to 120 min of SPT, respectively (*p* < 0.01), whereas the total tract digestible starch (TDSt) gradually decreased from 321 to 182 g/kg DM based on SPT time (*p* < 0.01). On the other hand, the truly digested protein in the small intestine (DVE) and the total metabolizable protein (MP) increased from 138 to 282 g/kg DM and 129 to 282 g/kg DM, respectively, with 0 to 120 min of SPT (quadratic, *p* < 0.01). The Feed Milk Value (FMV), based on both the DVE/OEB and NRC dairy nutrition systems, also increased with SPT (Quadratic, *p* < 0.01). The processing of FBS with SPT at 121 °C effectively reduced the highly degradable protein fraction in the rumen, shifting to a higher rumen undegraded protein (RUP) which was able to reach the small intestine. In the current study, the total MP, DVE, and FMV in dairy cows showed an overall increase with SPT in FBS.

## 1. Introduction

Feed types and heating methods are factors that impact the response of feedstuffs to heat processing. Steam pressure toasting (SPT) has been applied to modify the nutrient degradability and enhance the nutritional quality of pulses. In ruminants, the synchronized microbial fermentation of nutrients in the rumen and post-ruminal nutrient bioavailability are essential for adequate diet balancing and nutrient supply. The method of feed processing also impacts rumen fermentation and intestinal digestion.

Approximately 80% of faba bean (*Vicia faba*) protein is rapidly degraded in the rumen [1,2,3,4]. A high level of protein solubility has been demonstrated by a high rate and extent of protein disappearance after ruminal incubation in fistulated dairy cows and goats [5,6,7]. Rumen degradable protein (RDP) is the primary source of nitrogen for microbial utilization and growth. Rumen microbes and undegraded feed protein flow to the small intestine, contributing to the metabolizable protein pool [8]. However, how feed processing impacts metabolizable protein in faba bean seeds is still unclear.

Heat processing in pulses has been widely used to increase the nutritional value of the seeds by reducing detrimental components and modifying the nutrient degradation characteristics. As heating through dry or moist procedures alters the physical, chemical, and molecular properties of feedstuffs, steam pressure toasting (SPT) has been used to modify the nutrient degradability and degradation parameters in FBS to improve or balance the fermentation and digestion of feed nutrients in livestock animals [8,9]. The type, technique, and intensity of the processing are factors to be considered when determining the grade of response in terms of nutrient degradability and digestibility [2,9,10]. Based on previous research on the effects of SPT in peas and faba beans [2,9], the objective of this study was to evaluate the effects of SPT duration on in vitro intestinal digestibility and microbial protein synthesis (MCP), truly digested protein in the small intestine (DVE, MP), degraded protein balance (DPB), and predicted milk production performance (FMV) in dairy cows using the NRC_2001 and Dutch systems. The hypothesis of this study was that SPT would impact the nutritional values, which could be increased with increasing SPT duration.

## 2. Materials and Methods

All animal procedures and protocols used in this experiment were reviewed and approved by the University of Saskatchewan Animal Research Ethics Board (AREB), Animal Use Protocol (AUP #19910012), under the guidelines of the Canadian Council on Animal Care [11].

### 2.1. Samples and Sample Preparation

Snowbird variety faba bean seeds (FBS) from three years of harvest were obtained from the Crop Development Center (CDC), University of Saskatchewan, Saskatoon, SK. The seeds were evenly distributed (800 g) into aluminum trays (3.8 cm × 12.7 cm) and heated by SPT without a cover at 121 °C for 0, 30, 60, 90, and 120 min using a medium steam sterilizer autoclave (Amsco^®^ CenturyTM, STERIS Corporation, Mentor, OH, USA). The process included conditioning, sterilization (30, 60, 90, or 120 min), and exhaustion steps. The pressure inside the chamber during the sterilization period was around 17 psig, and the temperature ranged between a maximum of 123 and a minimum of 121 °C. A whole cycle process was initiated for every time point. At the end of the process, the trays were removed from the autoclave and cooled at ambient temperature. The detailed chemical composition profiles of the different treatments were reported previously [12,13].

### 2.2. Intestinal Digestion of Rumen Undegraded Protein

The intestinal digestibility of rumen undegraded protein (RUP) was determined in vitro using 12 h in situ feed rumen incubation residue samples following the three-step in vitro technique proposed by [14] and modified by [15]. The pre-incubation at 12 h with in situ nylon bag technique was applied using four rumen cannulated Holstein Friesian cows at the Rayner Dairy Teaching and Research Facilities, University of Saskatchewan. Details regarding the animals, their diet, and the in situ nylon bag procedure were reported previously [12,13]. In brief, solutions were prepared, and 0.35 g was weighed into 50 mL centrifuge tubes (duplicates) the same morning of the analysis. Then, 10 mL of pepsin (Sigma P-7012) solution 0.1 mol/L HCl (pH = 1.9) was added to every tube, vortexed, and incubated for one hour at 38 °C in a shaking water bath (Precision, Serial No: 602071249, Thermo Scientific, Waltham, MA, USA). After incubation, 0.5 mL (1 mol/L) NaOH solution and 13.5 mL of pancreatin (Sigma P-7545; pH = 7.8) were added to the tubes, vortexed, and incubated in the water bath for 24 h at 38 °C (shaker on). The tubes were vortexed three times during the 24 h period and at the end of incubation. Then, 3 mL of trichloroacetic acid (TCA) was added to stop the enzymatic hydrolysis; the mixture was then vortexed and kept at room temperature for 15 min before centrifuging for 15 min at 10,000× *g*. After centrifuging, 5 mL of supernatant was collected and analyzed for soluble N following the Kjeldahl method (AOAC 984.13). The protein intestinal digestibility was estimated as TCA-soluble N divided by the amount of N in the rumen-incubation residual sample.

### 2.3. Prediction of the Truly Digestible Protein Supply to the Small Intestine of Steam-Pressure Toasted Faba Bean Seeds in Dairy Cattle

The values from the in situ rumen degradation [12,13] and in vitro intestinal digestibility experiments were used to predict the true protein supply (DVE and MP) to dairy cattle using the DVE/OEB and NRC models. The DVE was estimated to account for the digestible feed protein, the microbial protein, and an endogenous protein loss correction. This value is equivalent to MP in the NRC model, with the difference that endogenous protein is included in the latter without any loss correction in the model.

### 2.4. Statistical Analysis

Data were statistically analyzed with a randomized complete block design (RCBD) and a mixed model, i.e., Y_ijk_ = μ + ρ_i_ + α_j_ + e_ijk_, using SAS software 9.4 (SAS Institute, Inc., Cary, NC, USA). Y_ijk_ was the observation of dependent variable ijk, μ was the population mean for the variable, ρ_i_ was the treatment effect (as a fixed effect), α_j_ was the in situ animals/run (as a random effect), and e_ijk_ was the random error associated with observation ij. A multiple comparison analysis with the Tukey method was used to compare the experimental treatments, and orthogonal polynomial contrast was used to determine the linear and quadratic effects of increasing SPT duration. Model assumptions were checked using residual analysis, and a normality test was performed using Procedure Univariate with Normal and Plot options. Significance was declared at *p* < 0.05 and trends at 0.05 ≤ *p* ≤ 0.10.

## 3. Results

### 3.1. Effect of Steam Pressure Toasting Duration on the In Vitro Intestinal Digestibility of Protein and Starch of Faba Bean Seeds

The crude protein (CP) digestibility (dIDP), the intestinally digested protein (IADP), and the rumen undegraded CP (RUP) showed a quadratic response to SPT and were higher with 120 min treatment than with other treatment durations (98.9%, 220 g/kg DM, 770 g/kg CP) (*p* < 0.05; Table 1). On the other hand, the total digested protein (TDP) was lower for 30, 60, and 90 min SPT (966, 962, and 964 g/kg CP, respectively) compared to 120 and 0 min SPT (992 and 995 g/kg CP) (Quadratic *p* = 0.01). The intestinal digestible starch (IDBSt) showed a quadratic response to SPT (*p* = 0.04), and 30, 60, and 90 min SPT treatments showed higher values (130, 104, and 91 g/kg DM) than 0 and 120 min SPT (84 and 53 g/kg DM, respectively). Moreover, the total tract digested starch (TDSt) linearly reduced with SPT duration from 0 to 120 min (*p* < 0.01).

### 3.2. Effect of Steam Pressure Toasting Duration on the Metabolic Characteristics, True Protein Supply, and Predicted Production Performance of Faba Bean Seeds

The fermentable organic matter (FOM) was reduced with SPT from 756 at 0 min to 418 g/kg DM at 120 min (Table 2), and it had a quadratic response to SPT duration (*p* < 0.01). Hence, the microbial protein synthesized in the rumen based on available energy (MREE) decreased with SPT and showed a quadratic response over time (*p* < 0.01). The rumen-synthesized microbial protein digested in the small intestine (DVME) decreased with SPT duration from 72 to 40 g/kg DM with 0 to 120 min SPT (Quadratic *p* < 0.01). On the other hand, the truly absorbed RUP in the small intestine (DVBE) increased from 68 (0 min) to 244 (120 min) g/kg DM (Quadratic *p* < 0.01). In the end, the truly digested protein in the small intestine (DVE) was higher for 120 min SPT than 60, 30, and 0 min (Quadratic *p* < 0.01); this was reflected by the same response in milk production performance (FMV) for the same treatments (Quadratic *p* < 0.01). The FMV increased from 2.81 to 5.73 kg milk/kg feed DM. Similar outcomes were observed for the metabolic characteristics estimated with NRC_2001 (Table 3).

## 4. Discussion

Post-ruminal absorption of feed nutrients is essential to determine the nutritional quality of feeds and the effects of feed processing. The purpose of decreasing protein degradation in the rumen is to enhance the amount of undegraded protein reaching the small intestine; therefore, besides solubility, the evaluation of protein digestibility in heated feeds is also essential [9,16]. Native proteins can be damaged during heat processing, raising concerns about the potential negative impact of this process on total tract protein digestibility [17]. Our obtained protein digestibility values in FBS partially agreed with the ones reported by [17]; those authors found a lower CP disappearance and higher RUP but no effects on the total tract protein digestibility. In the present study, SPT duration induced decreased protein digestibility with up to 90 min of heating with SPT; this was reflected by the same response on the total digested crude protein value based on g/kg CP. Even so, the protein digestibility and the whole tract digested protein were higher with the application of SPT for 120 min. Several authors have suggested that the reduced protein digestibility observed after heat processing is related to the severity of reactions occurring during heating [9,18]. Hence, the denaturation of the protein structure, the Maillard reaction, the development of Maillard products, the formation of cross-linkages to carbohydrates, and protein structure stabilization play a vital role in modifying protein solubility and degradation.

Nevertheless, the total protein digestibility might be impaired when permanent linkages and indigestible N are produced, reducing the protein availability in the small intestine [9,17,19]. Besides the physical and chemical changes produced by heating treatments, the nutritional quality of feeds can be enhanced by destroying or decreasing antinutritional compounds that interfere with the digestibility of nutrients [20]. According to [17], the reduction of tannins and trypsin inhibitors has proved beneficial in increasing in vitro protein digestibility in beans. In contrast, the concentrations of phytic acid, tannins, vicine, convicine, and trypsin inhibitors in FBS have been found to be reduced after autoclaving, with positive effects on protein digestibility [21,22,23]. It is worth noting that the faba bean variety used in the current study is characterized by a low or zero content of condensed tannins. However, the concentrations of anti-nutritional factors in the samples were not measured in this experiment.

Based on metabolic characteristics, the quantity of microbial protein synthesized in the rumen was reduced, while the amount of truly digested protein in the small intestine increased with SPT duration. These results might be associated with the increased quantity of truly absorbed RUP in the small intestine (DVBE) that was observed in the heated FBS, as DVE was computed as DVE = (DVME + DVBE) − DVMFE, where DVME was the rumen synthesized microbial protein digested in the small intestine and DVMFE the endogenous protein that was lost in digestion. Moreover, the predicted milk production performance (FMV) increased with SPT. These results could be related to the higher DVE, as well as the higher content of DVE directed for milk protein that was observed in the heated FBS. Previous research has demonstrated important associations between DVE and OEB values and the nutritional value of feed [9,16,18]. For instance, [18] reported increased DVE and reduced OEB values after roasting whole lupins and whole FBS, suggesting that a higher level of protein digestion was occurring in the small intestine than in the rumen. Similar results were observed in the current study. It is worth mentioning that OEB values close to zero are an indicator of adequate N balance in the rumen. Negative OEB values could represent a nitrogen deficiency that might impair microbial protein synthesis; conversely, greater values show significant nitrogen losses from the rumen [9,16,18]. In the current study, SPT changed the predicted true protein supplied to the small intestine and the predicted production performance in FBS. The DVE/MP and FMV gradually increased with increasing SPT duration, while lower nitrogen loss and shortage in the rumen were observed as SPT time increased.

## 5. Conclusions

Based on the current research, SPT increases the nutrient values of FBS in terms of total metabolizable protein (MP), truly absorbed nutrient supply (DVE), and feed milk value (FMV). Heating FBS between 60 and 90 min at 121 °C by steam pressure toasting might be optimal to achieve an adequate protein balance and predicted milk production performance in lactating dairy cows.

## Figures and Tables

**Table 1:** Effect of steam pressure toasting duration on the nutrient in vitro intestinal digestibility of Faba bean seeds.

Item	Raw	Steam Pressure Toasting Time (min)				SEM	*p* Value		Polynomial Contrast (*p* Value)	
		30	60	90	120			Raw vs. SPT	Linear	Quadratic
Dry matter digestibility, %										
dBDM	59.9 ^a^	51.9 ^a^	31.5 ^b^	25.9 ^b^	19.5 ^b^	3.02	<0.01	<0.01	<0.01	0.10
Intestinally digested DM, g/kg DM										
IDBDM	184 ^b^	239 ^a^	177 ^b^	153 ^bc^	125 ^c^	10.2	<0.01	0.32	<0.01	0.01
TDDM	876 ^a^	778 ^b^	613 ^c^	561 ^c^	482 ^d^	17.7	<0.01	<0.01	<0.01	0.02
Crude protein digestibility, %										
dIDP	97.8	92.8	94.1	94.9	98.9	1.63	0.13	0.19	0.42	0.02
Intestinally digested CP, g/kg DM										
IADP	62 ^d^	125 ^c^	179 ^b^	199 ^ab^	220 ^a^	7.4	<0.01	<0.01	<0.01	<0.01
TDP	285	281	284	285	287	7.3	0.73	0.79	0.46	0.43
Intestinally digested CP, g/kg CP										
RUP	220 ^d^	464 ^c^	646 ^b^	710 ^ab^	770 ^a^	23.0	<0.01	<0.01	<0.01	<0.01
IADP	215 ^d^	430 ^c^	608 ^b^	674 ^ab^	762 ^a^	21.2	<0.01	<0.01	<0.01	<0.01
EDCP	780 ^a^	536 ^b^	354 ^c^	290 ^cd^	230 ^d^	23.0	<0.01	<0.01	<0.01	<0.01
TDP	995	966	962	964	992	10.3	0.11	0.06	0.79	0.01
Starch digestibility, %										
dBSt	82.8 ^a^	68.4 ^a^	41.0 ^b^	35.3 ^b^	19.9 ^b^	5.70	<0.01	<0.01	<0.01	0.38
Intestinally digested starch, %St										
IDBSt	23.2 ^ab^	33.9 ^a^	22.9 ^ab^	21.2 ^ab^	12.8 ^b^	3.21	0.01	0.9	0.01	0.04
TDSt	94.8 ^a^	84.3 ^b^	68.5 ^c^	61.4 ^c^	49.1 ^d^	2.24	<0.01	<0.01	<0.01	0.57
Intestinally digested starch, g/kg DM										
IDBSt	84.4	130	104	90.9	52.8	16.98	0.09	0.61	0.09	0.04
TDSt	321 ^a^	303 ^ab^	288 ^ab^	247 ^ab^	182 ^b^	28.9	0.04	0.07	<0.01	0.29

^a–d^ Means with different letters in the same row are significantly different (*p* < 0.05). Samples: Faba bean seeds, Snowbird variety from 3 years of harvest. Control treatment: raw seeds. Heating process: steam pressure toasting at 121 °C for 30, 60, 90, and 120 min. dBDM, intestinal digestibility of rumen bypass dry matter; dIDP, intestinal digestibility of rumen bypass protein; IADP, intestinal digestible crude protein; IDBDM, intestinally digested rumen bypass dry matter; TDDM, total digested dry matter; TDP, total digested crude protein; RUP, rumen undegradable crude protein; EDCP, effective degradable crude protein; dBSt, intestinal digestibility of rumen undegraded starch; IDBSt, intestinally digested rumen undegraded starch; TDSt, total digestible starch. SEM, Standard Error of Mean. Multi-treatment comparisons using the Tukey method.

**Table 2:** Effect of steam pressure toasting duration on the metabolic characteristics, true protein supply, and predicted production performance of Faba bean seeds based on the Dutch system (DVE/OEB).

Item	Raw	Steam Pressure Toasting Time (min)				SEM	*p* Value		Polynomial Contrast (*p* Value)	
		30	60	90	120			Raw vs. SPT	Linear	Quadratic
Metabolic characteristics, g/kg DM										
DOM	944	947	951	931	939	5.1	0.13	0.67	0.13	0.52
UDOM	13.5 ^a^	11.7 ^ab^	10.4 ^b^	11.3 ^b^	10.9 ^b^	0.60	<0.01	<0.01	<0.01	0.01
DASH	27.3	26.7	25.2	37.5	32.4	3.14	0.11	0.38	0.06	0.69
UASH	14.7	14.4	13.6	20.2	17.4	1.69	0.11	0.38	0.06	0.69
UDM	28.2	26.1	23.9	31.5	28.3	2.01	0.15	0.73	0.37	0.30
FOM	756 ^a^	585 ^b^	475 ^c^	420 ^c^	418 ^c^	24.4	<0.01	<0.01	<0.01	<0.01
MREE	113 ^a^	87.7 ^b^	71.2 ^c^	63.0 ^c^	62.8 ^c^	3.65	<0.01	<0.01	<0.01	<0.01
DVME	72.3 ^a^	55.9 ^b^	45.4 ^c^	40.2 ^c^	40.0 ^c^	2.33	<0.01	<0.01	<0.01	<0.01
DVMFE	2.12	1.96	1.80	2.36	2.13	0.151	0.15	0.73	0.37	0.30
DVBE	68.4 ^d^	139 ^c^	199 ^b^	221 ^ab^	244 ^a^	8.2	<0.01	<0.01	<0.01	<0.01
MREN	217 ^a^	140 ^b^	83.6 ^c^	62.6 ^cd^	42.1 ^d^	7.12	<0.01	<0.01	<0.01	<0.01
Protein supply into the small intestine, g/kg DM										
TPSI	155 ^d^	216 ^c^	265 ^b^	281 ^ab^	294 ^a^	7.5	<0.01	<0.01	<0.01	<0.01
DVE	138 ^d^	193 ^c^	243 ^b^	259 ^ab^	282 ^a^	7.3	<0.01	<0.01	<0.01	<0.01
Degraded protein balance	103 ^a^	52.8 ^b^	12.4 ^c^	−0.42 ^cd^	−20.7 ^d^	5.89	<0.01	<0.01	<0.01	<0.01
Predicted milk production performance/FMV										
DVE for milk protein, g/kg DM	92.8 ^d^	129 ^c^	163 ^b^	174 ^ab^	189 ^a^	4.9	<0.01	<0.01	<0.01	<0.01
FMV, kg milk/kg DM feed	2.81 ^d^	3.92 ^c^	4.93 ^b^	5.26 ^ab^	5.73 ^a^	0.148	<0.01	<0.01	<0.01	<0.01

^a–d^ Means with different letters in the same row are significantly different (*p* < 0.05). Samples: Faba bean seeds, Snowbird variety from 3 years of harvest. Control treatment: raw seeds. Heating process: steam pressure toasting at 121 °C for 30, 60, 90, and 120 min. DOM, digested organic matter; UASH, undigestible inorganic matter; UDM, undigestible DM (indigestible organic matter + indigestible inorganic matter); UDOM, undigested organic matter; DASH, digestible inorganic matter; DVBE, truly absorbed bypass protein in the small intestine; DVE, truly digested protein in the small intestine computed as (DVME + DVBE) − DVMFE; DVME, rumen synthesized microbial protein digested in the small intestine; DVMFE, endogenous protein losses in digestion; FMV, feed milk value, computed based on DVE; FOM, fermentable organic matter; MREE-MCP_fom_, microbial protein synthesized in the rumen based on available energy; MREN, microbial protein synthesized in the rumen; OEB, degraded protein balance; TPSI, true protein supplied to the small intestine. SEM, Standard Error of Mean. Multi-treatment comparisons using the Tukey method. The FMV for the current samples was computed as FMV = DVE for milk protein/33, where the protein composition in the milk was assumed to be 33 g protein/1000 g milk. Additionally, the DVE for milk production was obtained by assuming an efficiency of metabolizable protein for lactation of 0.67, multiplied by the total DVE content found in FBS.

**Table 3:** Effect of steam pressure toasting duration on the metabolic characteristics, true protein supply, and predicted production performance of Faba bean seeds based on the NRC dairy model.

Item	Raw	Steam Pressure Toasting Time (min)				SEM	*p* Value	Contrast (*p* Value)	Polynomial Contrast (*p* Value)	
		30	60	90	120			Raw vs. SPT	Linear	Quadratic
Metabolic characteristics, g/kg DM										
TDN_3×_	764 ^a^	739 ^ab^	729 ^bc^	705 ^cd^	693 ^d^	8.1	<0.01	<0.01	<0.01	0.59
MCP_RDP_	190 ^a^	132 ^b^	88.9 ^c^	72.9 ^cd^	56.6 ^d^	5.62	<0.01	<0.01	<0.01	<0.01
MCP_TDN_	99.3 ^a^	96.0 ^ab^	94.7 ^bc^	91.7 ^cd^	90.1 ^d^	1.05	<0.01	<0.01	<0.01	0.59
AMCP	63.6 ^a^	61.5 ^ab^	60.6 ^bc^	58.7 ^cd^	57.7 ^d^	0.67	<0.01	<0.01	<0.01	0.59
RUP	63.1 ^d^	135 ^c^	191 ^b^	210 ^ab^	223 ^a^	8.34	<0.01	<0.01	<0.01	<0.01
ARUP	61.6 ^d^	125 ^c^	179 ^b^	199 ^ab^	220 ^a^	7.43	<0.01	<0.01	<0.01	<0.01
ECP	10.9 ^a^	10.8 ^ab^	10.7 ^bc^	10.7 ^c^	10.8 ^abc^	0.05	0.01	<0.01	<0.01	0.01
AECP	4.35 ^a^	4.32 ^ab^	4.30 ^bc^	4.27 ^c^	4.30 ^abc^	0.019	0.01	<0.01	<0.01	0.01
Protein supply into the small intestine, g/kg DM										
Total MP	129 ^d^	191 ^c^	244 ^b^	262 ^ab^	282 ^a^	7.9	<0.01	<0.01	<0.01	<0.01
Degraded protein balance	106 ^a^	42.1 ^b^	−7.19 ^c^	−22.4 ^cd^	−39.8 ^d^	6.83	<0.01	<0.01	<0.01	<0.01
Predicted milk production performance/FMV										
MP for milk protein, g/kg DM	86.8 ^d^	128 ^c^	164 ^b^	176 ^ab^	189 ^a^	5.28	<0.01	<0.01	<0.01	<0.01
FMV, kg milk/kg DM feed	2.63 ^d^	3.88 ^c^	4.96 ^b^	5.33 ^ab^	5.73 ^a^	0.160	<0.01	<0.01	<0.01	<0.01

^a–d^ Means with different letters in the same row are significantly different (*p* < 0.05). Samples: Faba bean seeds, Snowbird variety from 3 years of harvest. Control treatment: raw seeds. Heating process: SPT, steam pressure toasting at 121 °C for 30, 60, 90, and 120 min. TDN_3×_, total digestible nutrient at the production level (3× maintenance intake); AECP, truly absorbed rumen endogenous protein in the small intestine; AMCP, truly absorbed microbial protein in the small intestine; ARUP, truly absorbed rumen undegradable protein in the small intestine; DPB, rumen degraded protein balance; ECP, rumen endogenous protein; FMV, feed milk value, computed based on MP; MCP_RDP_, microbial protein synthesized in the rumen based on rumen degradable protein; TDN, total digestible nutrients; MCP_TDN_, rumen synthesized microbial protein base on available TDN; MP, metabolizable protein; SEM, Standard Error of Mean. Multi-treatment comparisons using the Tukey method.

## Data Availability

Data are contained within the article.
